# Capacitively Coupled CSRR and H-Slot UHF RFID Antenna for Wireless Glucose Concentration Monitoring

**DOI:** 10.3390/s25185651

**Published:** 2025-09-10

**Authors:** Tauseef Hussain, Jamal Abounasr, Ignacio Gil, Raúl Fernández-García

**Affiliations:** Department of Electronic Engineering, Universitat Politècnica de Catalunya, 08222 Terrassa, Spain; jamal.abounasr@upc.edu (J.A.); ignasi.gil@upc.edu (I.G.); raul.fernandez-garcia@upc.edu (R.F.-G.)

**Keywords:** RFID glucose sensor, complementary split-ring resonator (CSRR), dielectric sensing, passive wireless sensing, resonator-based sensing, concentration monitoring

## Abstract

This paper presents a fully passive and wireless glucose concentration sensor that integrates a capacitively coupled complementary split-ring resonator (CSRR) with an H-slot UHF RFID antenna. The CSRR serves as the primary sensing element, where changes in glucose concentration alter the effective permittivity of the surrounding solution, thereby modifying the resonator capacitance and shifting its resonance behavior. Through near-field capacitive coupling, these dielectric variations affect the antenna input impedance and backscatter response, enabling wireless sensing by modulating the maximum read range. The proposed sensor operates within the 902–928 MHz UHF RFID band and is interrogated using commercial RFID readers, eliminating the need for specialized laboratory equipment such as vector network analyzers. Full-wave electromagnetic simulations and experimental measurements validate the sensor performance, demonstrating a variation in the read range from 6.23 m to 4.67 m as glucose concentration increases from 50 to 200 mg/dL. Moreover, the sensor exhibits excellent linearity, with a high coefficient of determination ($R^{2}=0.986$) based on the curve-fitted data. These results underscore the feasibility of the proposed sensor as a low-cost and fully portable platform for concentration monitoring, with potential applications in liquid characterization and chemical sensing.

## 1. Introduction

Microwave dielectric sensors have emerged as a promising platform for material and liquid characterization due to their ability to detect small changes in permittivity. The dielectric constant of a material provides key insights into its molecular composition and concentration, making it a valuable parameter for quality control and sensing applications in diverse fields such as agriculture, pharmaceuticals, and food processing [1,2,3]. Compared to conventional analytical techniques such as refractometry, chemical titration, or spectroscopy, microwave planar resonator-based sensors offer advantages including non-intrusive operation, compactness, low cost, and compatibility with planar fabrication technologies [4,5,6,7]. Among these, complementary split-ring resonators (CSRRs) have gained particular interest due to their strong electric field localization, high quality factor, and compact size. These properties make CSRRs especially suitable for detecting subtle permittivity variations in both liquids and solid materials [8,9,10].

Accordingly, various CSRR topologies have been explored specifically for glucose concentration monitoring applications. These include conventional single- and dual-ring structures [11,12,13], triple-pole resonators offering enhanced field localization [14,15], and honeycomb-shaped arrays designed to increase surface area and improve sensitivity [16]. To further optimize performance, researchers have proposed advanced geometries such as hexagonal and triangular layouts [17,18], interdigital-capacitor-loaded configurations [19], and double-square or concentric multiband structures [20,21,22], which introduce multiple resonance behaviors and extend the detection range. In addition to geometrical innovations, robustness under varying operating conditions has been addressed through temperature-compensated designs [23] and switching-circuit integration [24] in order to mitigate baseline environmental drift and improve the sensing accuracy.

Such CSRR-based dielectric sensors are typically excited through microstrip lines, striplines, or open-ended transmission lines. Meanwhile, in some configurations, patch antennas are used to couple energy into the resonant structure. The sensing principle relies on shifts in resonant frequency or scattering parameters caused by dielectric perturbations from the material under test [25,26,27]. Since CSRR-based sensors typically operate at microwave frequencies between 1 and 6 GHz, and in some instances up to 25 GHz, most systems require the use of vector network analyzers (VNAs) or specialized RF instrumentation to obtain sensing responses through measurements of reflection (S_11_) or transmission (S_21_) coefficients [28,29,30,31,32]. Although these designs demonstrate a strong sensing performance, their reliance on laboratory-grade equipment remains a significant barrier to portability and cost-effectiveness, thereby hindering their practical deployment.

Recently, passive UHF RFID technology has shown strong potential for wireless, battery-free, and portable sensing applications [33]. In RFID-based systems, changes in the surrounding dielectric environment can alter the tag impedance or radiation characteristics, which are detectable through variations in backscattered signal strength or read range using commercial RFID readers [33]. This capability has enabled the development of RFID-enabled dielectric sensors for applications such as moisture detection, food quality assessment, and biochemical monitoring [34,35,36]. Similarly, various RFID-based sensors have been designed for liquid characterization and glucose concentration monitoring, often relying on textile-based absorbent materials [37,38] or dual-tag configurations [39]. However, despite the proven sensitivity of microwave resonators such as CSRRs in laboratory settings, their integration into passive UHF RFID platforms remains largely unexplored for the wireless and portable monitoring of solute concentrations in liquids.

In this work, we present the integration of a circular CSRR placed over an H-slot RFID antenna for the passive and wireless sensing of glucose concentration in aqueous solutions. The variations in glucose levels affect the permittivity of the solution placed above the resonator, which modifies the resonator capacitance and, through near-field coupling, affects the antenna input impedance and gain. Subsequently, these changes modulate the RFID backscatter response, which can be monitored through variations in the maximum read range using commercial UHF RFID readers operating in the 902–928 MHz band. Thus, by combining the high dielectric sensitivity of CSRRs, the proposed sensor offers a fully passive and portable platform for wireless glucose concentration monitoring. The following sections describe the sensor design methodology, simulations, fabrication, experimental validation, and a comparative analysis with existing CSRR-based glucose sensors.

## 2. Design Concept and Modeling

**Figure 1:**
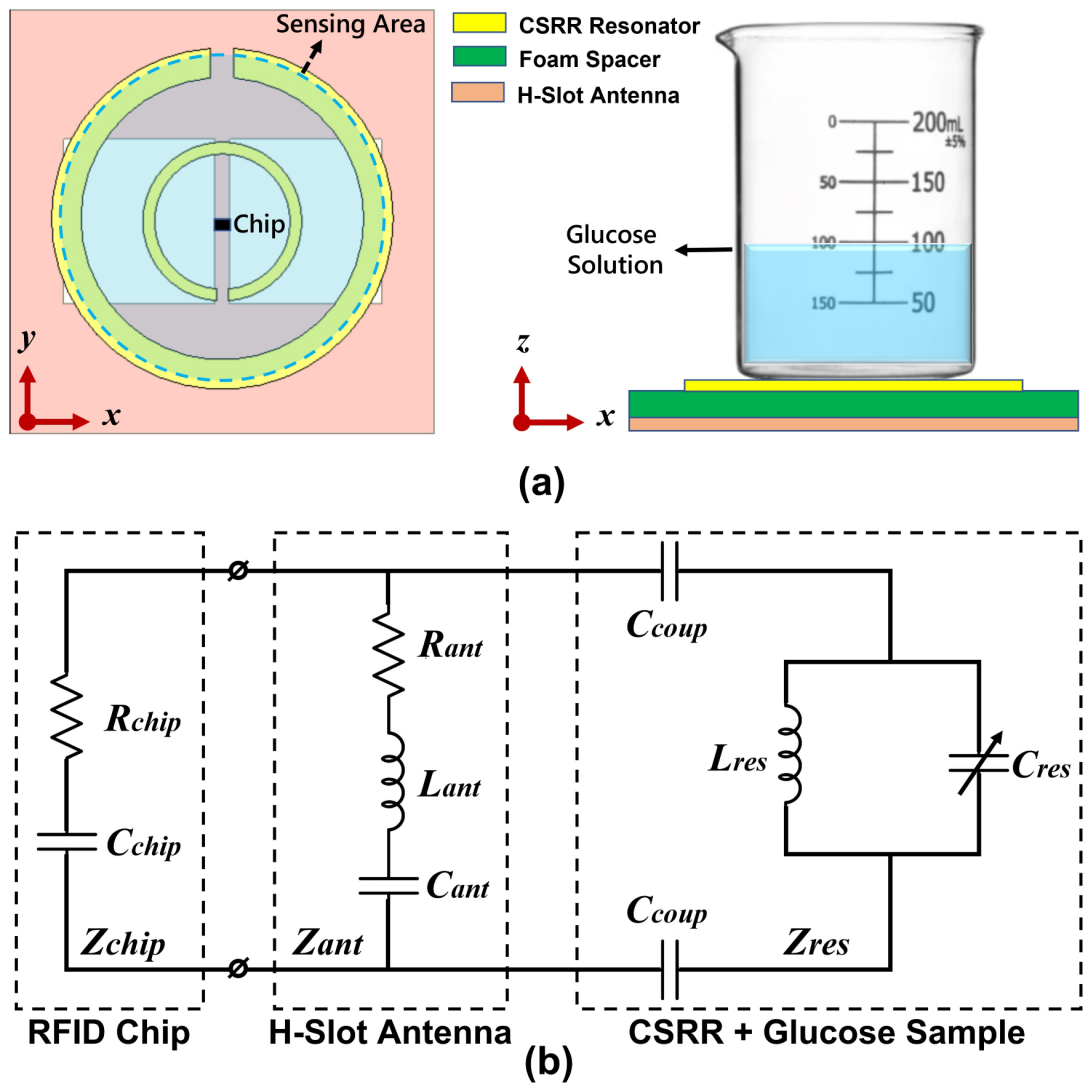
RFID-CSRR glucose sensor architecture, (**a**) Circular CSRR stacked above the H-slot antenna (**left**) and glucose container placed above the resonator sensing area (**right**). (**b**) Equivalent circuit model of the sensor with the glucose sample.

To achieve the sensing mechanism, the circular CSRR is stacked directly above the H-slot UHF RFID patch antenna, as illustrated in Figure 1a. When a container of glucose solution is placed over the sensing region, the resulting dielectric loading perturbs the resonance characteristics of the CSRR depending on the glucose concentration. Since the CSRR is capacitively coupled to the antenna, these perturbations influence its input impedance and radiation behavior, which, in turn, affects the power delivered to the RFID chip, thereby modulating the backscatter response. Consequently, glucose concentration levels can be estimated from the corresponding variations in maximum read range, measured using standard portable UHF RFID readers.

### 2.1. Dielectric Modeling of Glucose Solutions

At microwave frequencies, the dielectric properties of aqueous glucose solutions can be described by their complex permittivity, which depends on both angular frequency (ω) and glucose concentration (*c*); this is given as follows: [39]:

(1) $$\varepsilon^{*}\left(\omega ,c\right)=\varepsilon_{r}\left(\omega ,c\right)-j\varepsilon_{j}\left(\omega ,c\right)$$

where the real and imaginary parts, $\varepsilon_{r}$ and $\varepsilon_{j}$, represent the dielectric constant and dielectric losses, respectively. According to the first-order Debye relaxation model, $\varepsilon_{r}$ decreases with either increasing frequency or glucose concentration due to reduced dipolar relaxation and the displacement of water molecules [40]. Thus, in the proposed sensor, the resonant behavior of the CSRR is primarily governed by $\varepsilon_{r}$, which determines the effective capacitance of the resonator and leads to measurable shifts in its resonance characteristics.

### 2.2. Equivalent Circuit Model

An equivalent circuit model, shown in Figure 1b, is developed to analytically describe the sensing behavior of the proposed RFID-CSRR system. The slotted patch antenna is modeled as a series RLC circuit, while the CSRR is represented as a parallel LC tank. These two elements are capacitively coupled through the coupling capacitor ($C_{\mathrm{coup}}$), which represents the near-field interaction enabled by their vertical stacking. Thus, the total input impedance seen by the RFID chip is a combination of the antenna ($Z_{\mathrm{ant}}$) and the resonator ($Z_{\mathrm{res}}$), and can be expressed as follows:

(2) $$Z_{\mathrm{eff}}\left(c\right)=Z_{\mathrm{ant}}\left(c\right)+Z_{\mathrm{res}}\left(c\right)$$

where $Z_{\mathrm{res}}\left(c\right)$ is the equivalent impedance of the CSRR with the glucose sample and the coupling capacitance, which is given as follows:

(3) $$Z_{\mathrm{res}}\left(c\right)=\left(\frac{2}{j\omega C_{\mathrm{coup}}}+{\left(\frac{1}{j\omega C_{\mathrm{res}}\left(c\right)}+j\omega L_{\mathrm{res}}\right)}^{-1}\right)$$

During sensing, as glucose concentration increases, the effective permittivity around the CSRR decreases, leading to a reduction in its capacitance $C_{\mathrm{res}}\left(c\right)$. This change alters the resonator impedance $Z_{\mathrm{res}}\left(c\right)$ and, in turn, the overall input impedance $Z_{\mathrm{eff}}\left(c\right)$ seen by the chip. These variations impact the power transfer efficiency between the antenna and chip, which can be quantified by the power transmission coefficient as follows:

(4) τ(c)=4Re(Zeff(c))Re(Zchip)|Zeff(c)+Zchip|2

Additionally, the antenna gain $G_{\mathrm{tag}}\left(c\right)$ may also vary with the glucose concentrations due to changes in dielectric loading and current redistribution near the CSRR. Consequently, these combined effects influence the maximum read range of the RFID tag, which can be determined using the Friis transmission formula, as follows [41]:

(5) $$d_{\max}=\frac{\lambda }{4\pi }\sqrt{\frac{P_{t}G_{t}G_{\mathrm{tag}}\tau }{P_{\mathrm{chip}}}}$$

where $P_{t}$ represents the transmitted power; $G_{t}$ and $G_{\mathrm{tag}}$ are the gains of the reader and tag antennas, respectively; λ is the operating wavelength; and $P_{\mathrm{chip}}$ is the wake-up power threshold required for the RFID chip to function. This modeling framework establishes the foundation for the subsequent design optimization, fabrication, and experimental validation of the proposed RFID-CSRR-based glucose concentration sensor.

## 3. Sensor Design and Simulation

### 3.1. Antenna and Resonator Geometry

#### 3.1.1. H-Slot Antenna

**Figure 2:**
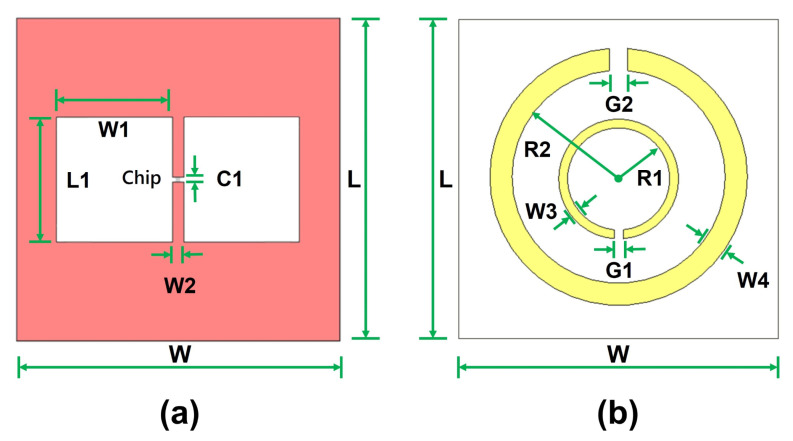
Geometry of the glucose sensing system. (**a**) H-slot UHF RFID antenna. (**b**) Complementary split-ring resonator (CSRR).

The proposed UHF RFID sensor employs a compact H-slot patch antenna that is specifically designed to operate within the upper European UHF RFID band (915–921 MHz), as shown in Figure 2a. The slot geometry was chosen to support broadband impedance matching and to enhance near-field capacitive coupling while maintaining a compact form factor. The antenna was initially optimized independently for the NXP UCODE 7xm RFID chip, which has an input impedance of approximately 17-j274 Ω at 915 MHz [42]. This chip was selected for its low activation threshold of −18.5 dBm, which can facilitate reliable performance even in high-dielectric aqueous environments. The preliminary antenna design resulted in a patch size of $72.3\;{\mathrm{mm}}^{2}$, with slot dimensions of $L_{1}=27.0\;\mathrm{mm}$ and $W_{1}=25.2\;\mathrm{mm}$, achieving an approximate conjugate impedance matching with the RFID chip in the standalone configuration.

#### 3.1.2. Circular CSRR

A circular complementary split-ring resonator (CSRR) was subsequently designed, as shown in Figure 2b, for integration above the slot antenna. The inner ring was dimensioned to fit precisely within the length of the slot aperture to promote strong capacitive coupling, and its open ends were oriented toward the feedline to ensure effective excitation, as illustrated in Figure 1a. Moreover, the outer ring was sized to approximately match the 50 mm diameter of the sample-container base, ensuring that the resonator’s electric field uniformly covers the cross-sectional area of the sensing medium. As expected, this integration introduced additional reactive loading, which perturbed the antenna impedance. Therefore, the slot width ($W_{1}$) was re-optimized while maintaining the slot length ($L_{1}$) to achieve conjugate matching with the RFID chip. The optimized dimensions of the antenna and CSRR as an integrated unit are summarized in Table 1.

### 3.2. Simulation Setup

Full-wave electromagnetic simulations were conducted in CST Studio Suite 2024 to evaluate the sensor performance. The time-domain solver with hexahedral meshing was employed for the accurate modeling of broadband and near-field interactions. A discrete port was placed across the chip terminals to emulate the RFID interface, while open boundary conditions were applied to approximate free-space operation. The H-slot antenna was modeled on a 125 μm PET substrate ($\varepsilon_{r}=3.2$, tanδ=0.005), while the CSRR was patterned on a 200 μm polyimide (PI) layer ($\varepsilon_{r}=3.5$, tanδ=0.003). A 1.5 mm thick air spacer was inserted between the two layers to ensure electrical isolation.

Furthermore, to simulate the sensing medium, the glucose solution was modeled as a dielectric cylinder placed inside a 1 mm thick PET container ($\varepsilon_{r}=3.2$) positioned directly above the sensing region of the CSRR. The dielectric constants were extracted from experimental data reported in [43], estimated using the Debye relaxation model, which shows a linear decrease in permittivity at 915 MHz with increasing glucose concentration. The simulation outputs included variations in antenna input impedance, gain, and power transmission coefficient across different glucose concentrations.

### 3.3. Simulation Results

#### 3.3.1. Antenna Impedance

**Figure 3:**
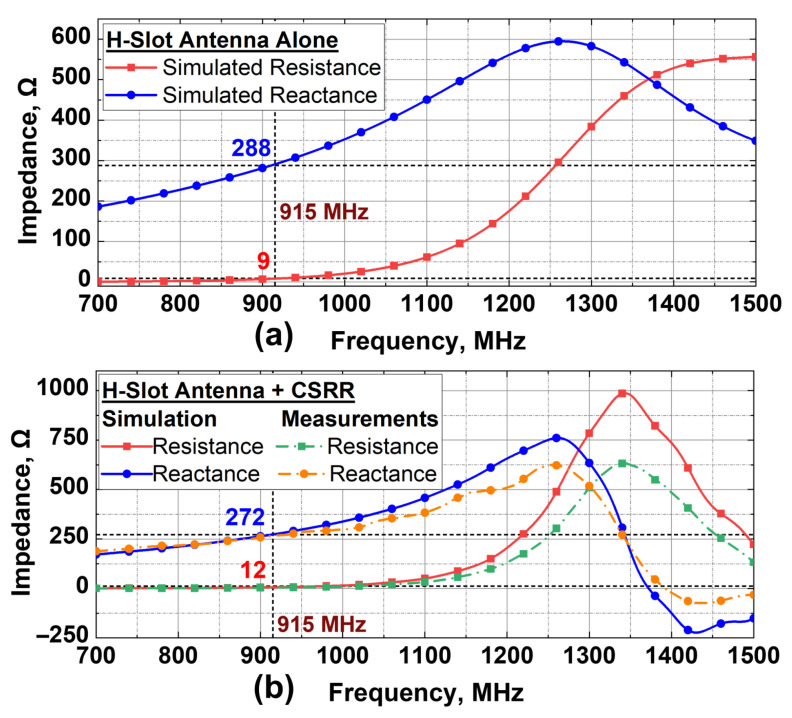
Simulated and measured antenna input impedance. (**a**) Standalone H-slot antenna. (**b**) H-slot antenna integrated with CSRR.

The input impedance of the H-slot antenna was first evaluated to assess the impact of its integration with the CSRR. As shown in Figure 3a, the standalone antenna with preliminary slot dimensions exhibited a resistance of 9 Ω and a reactance of 288 Ω at 915 MHz, indicating moderate impedance matching (τ≈0.70) with the RFID chip. Subsequently, after integrating the CSRR and re-optimizing the antenna slot dimensions, the resistance and reactance approached approximately 12 Ω and 272 Ω, respectively. The simulated and measured impedance responses after CSRR integration are presented in Figure 3b. This reduction in reactance can be attributed to the additional capacitive loading introduced by the CSRR, which partially compensates for the inherent inductive reactance of the antenna. As a result, the antenna impedance aligns more closely with the complex conjugate of the RFID chip, yielding a higher power transmission coefficient (τ≈0.96), which enhances power transfer efficiency and improves the read-range performance.

#### 3.3.2. Electric Field Distribution

**Figure 4:**
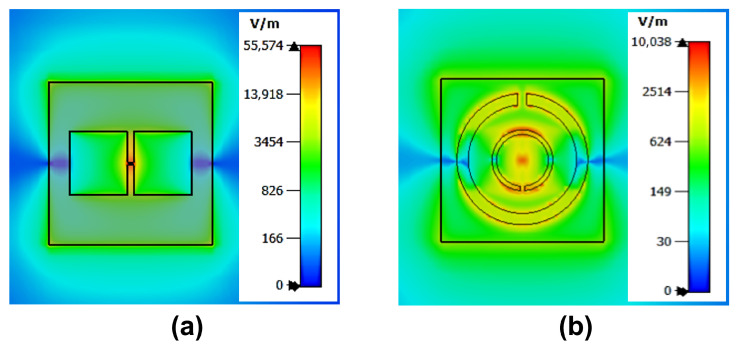
Simulated electric field distribution at 915 MHz. (**a**) Standalone H-slot antenna. (**b**) H-slot antenna integrated with CSRR.

The electric field distribution was further analyzed to evaluate the sensing area of the slot antenna, both with and without the integrated CSRR. As shown in Figure 4a, the standalone antenna exhibits a localized electric field concentrated near the feed point, indicating a limited sensing area just around the chip.

In contrast, Figure 4b shows the field distribution after CSRR integration. In this configuration, strong electric coupling occurs between the feedline and the inner ring of the CSRR, which subsequently excites the outer ring and results in field expansion across the entire resonator surface. This enhanced field distribution confirms that the integrated CSRR extends the effective sensing area, enabling more uniform interaction with the glucose solution inside the container, thereby improving sensitivity to the dielectric variations.

#### 3.3.3. Glucose Sensing Response

**Figure 5:**
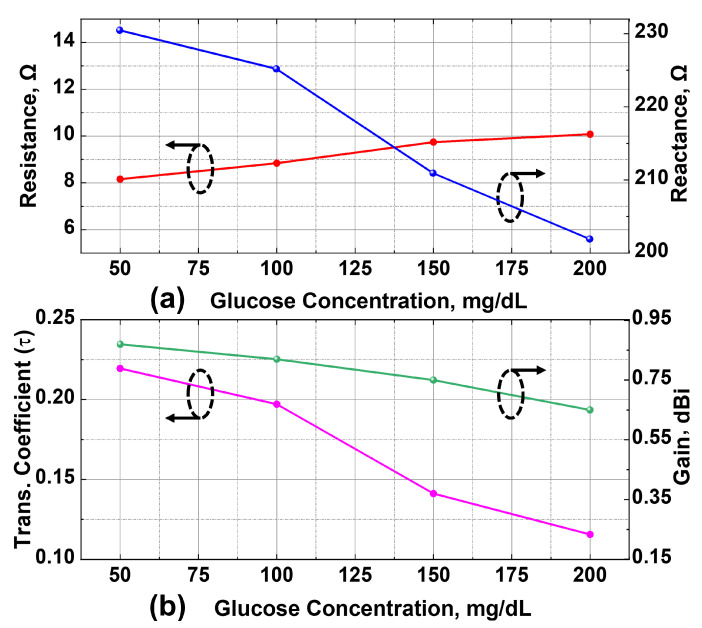
Simulated glucose sensing response of the proposed RFID-CSRR sensor at 915 MHz. (**a**) Input impedance variation with glucose concentration. (**b**) Corresponding variations in power transmission coefficient (τ) and antenna gain.

To evaluate the sensing capability of the proposed structure, simulations were performed with a dielectric cylinder representing the glucose solution positioned over the CSRR sensing area. As shown in Figure 5a, the presence of the aqueous solution significantly alters the antenna input impedance, primarily reducing its reactance due to increased capacitive loading, compared to the unloaded case, where the resistance and reactance were approximately 12Ω and 272Ω, respectively. This shift is attributed to the higher permittivity of water content ($\varepsilon_{r}\approx 78$), which increases the effective capacitance of the CSRR and consequently modifies the antenna input impedance. Beyond this, variations in the glucose concentration induce additional changes in the antenna impedance. Thus, as the concentration increases from 50 to 200 mg/dL, the reactance exhibits a consistent decrease from approximately 230Ω to 201Ω, while the resistance remains relatively stable, varying slightly between 8 and 10Ω. These impedance variations confirm that glucose-induced dielectric changes modulate the capacitive loading of the CSRR.

Moreover, Figure 5b presents the corresponding variations in the power transmission coefficient (τ) and antenna gain. The value of τ, which drops to approximately 0.22 in the presence of the aqueous glucose solution, continues to decline with increasing glucose concentration, indicating reduced impedance matching. Similarly, the antenna gain also exhibits a slight decrease with rising concentration, ranging from 0.87 dBi to 0.65 dBi. Together, these observed trends validate the theoretical model, which predicts that variations in τ and antenna gain would directly influence the read range. Therefore, these results demonstrate that glucose concentration can be effectively monitored by tracking variations in the maximum read range using a standard RFID reader setup.

## 4. Fabrication and Measurements

### 4.1. Fabrication Process

The proposed glucose sensor was fabricated using a hybrid fabrication approach to accommodate the distinct structural and functional requirements of the tag antenna and the CSRR. The H-slot antenna was fabricated by patterning adhesive copper foil onto a 125 µm thick PET substrate, which was selected for its low dielectric loss at UHF frequencies. The use of copper foil enabled rapid prototyping while providing low-resistance conductive paths suitable for RFID operation. Furthermore, to complete the tag design, the RFID chip was mounted at the feed point using silver conductive epoxy (Chemtronics, Kennesaw, GA, USA) to ensure reliable electrical contact.

Moreover, the CSRR was fabricated on a 200 μm thick polyimide (PI) substrate, which was chosen for its stable permittivity under varying temperature and humidity, which is advantageous for reliable sensing applications. The CSRR structure was printed using a NOVA DIW platform (Voltera, Waterloo, ON, Canada) with silver conductive ink ACI-FS0142A (ACI Materials, Goleta, CA, USA), achieving fine resolution suitable for the curved geometries of the resonator. The printed structure was then cured at 150 °C for 10 min to improve the conductivity and adhesion.

**Figure 6:**
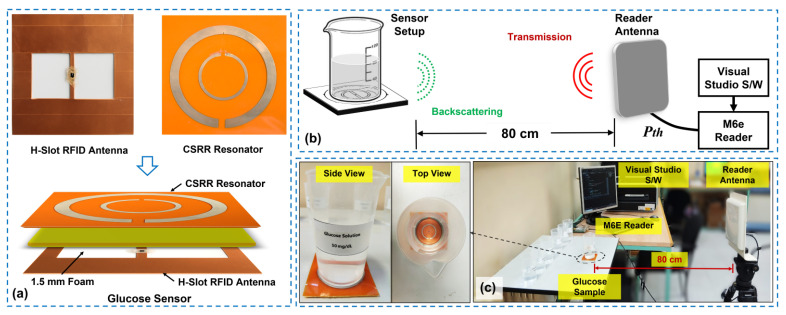
Fabrication and measurement of the proposed RFID-CSRR sensor. (**a**) Fabricated glucose sensor integrating the H-slot antenna and CSRR. (**b**) Schematic diagram of the RFID-based measurement setup. (**c**) Photograph of the experimental setup showing the RFID reader and sensor with the glucose sample.

In the final step, the tag antenna and CSRR layers were assembled in a vertically stacked configuration using a 1.5 mm thick foam spacer (Aearo Technologies, Indianapolis, IN, USA), forming the complete RFID-CSRR-based glucose concentration sensor, as shown at the bottom of Figure 6a.

### 4.2. Experimental Setup

The test samples of aqueous glucose solutions were prepared in identical PET containers by dissolving different amounts of analytical-grade D-glucose in 100 mL of deionized water to obtain concentrations ranging from 50 to 200 mg/dL. A precision digital mini-scale with a resolution of 1 mg was used to accurately weigh the glucose for solution preparation. During testing, each prepared container was positioned directly over the sensing area of the CSRR. Moreover, for concentration measurements, the sensor was interrogated remotely using a ThingMagic M6E RFID reader connected to a circularly polarized antenna (MT-242 025/TRH/A/A) via a low-loss coaxial cable. The schematic diagram of the proposed glucose concentration sensing system is shown in Figure 6b.

The measurements were based on estimating the minimum transmit power required to activate the tag at a fixed distance [44]. Accordingly, the reader antenna was positioned at a distance of 0.8 m from the sensor, as shown in Figure 6c. This represents a typical mid-range operational distance for UHF RFID systems and was found to provide reliable measurements in the lab settings while avoiding near-field coupling effects. Moreover, a custom software interface, developed using Microsoft Visual Studio 2022 (Community Edition), was employed to control the reader and perform automated power sweeps across the UHF RFID band. The software incrementally reduced the transmit power until the tag ceased to respond. Then, the minimum transmit power required to activate the RFID chip was recorded as threshold power ($P_{\mathrm{th}}$), and was used to derive key sensor performance metrics, including the $\tau G_{\mathrm{tag}}$ product and the maximum read range ($d_{\max}$).

### 4.3. Glucose Measurements

#### 4.3.1. Threshold Power Analysis

The fabricated RFID-CSRR glucose sensor was first experimentally characterized through threshold power measurements across the entire FCC-authorized UHF RFID band (902–928 MHz). The primary objective at this stage was to evaluate the electromagnetic behavior of the sensor over the wider frequency range. For each sample, the threshold power was recorded at eight frequency points. At each frequency, the transmit power from the reader was decreased from 20 dBm in 0.5 dBm steps until the tag no longer responded to consecutive RFID reader queries. The power step size was selected based on the resolution capabilities of the RFID reader and to maintain a practical balance between measurement precision and acquisition time.

**Figure 7:**
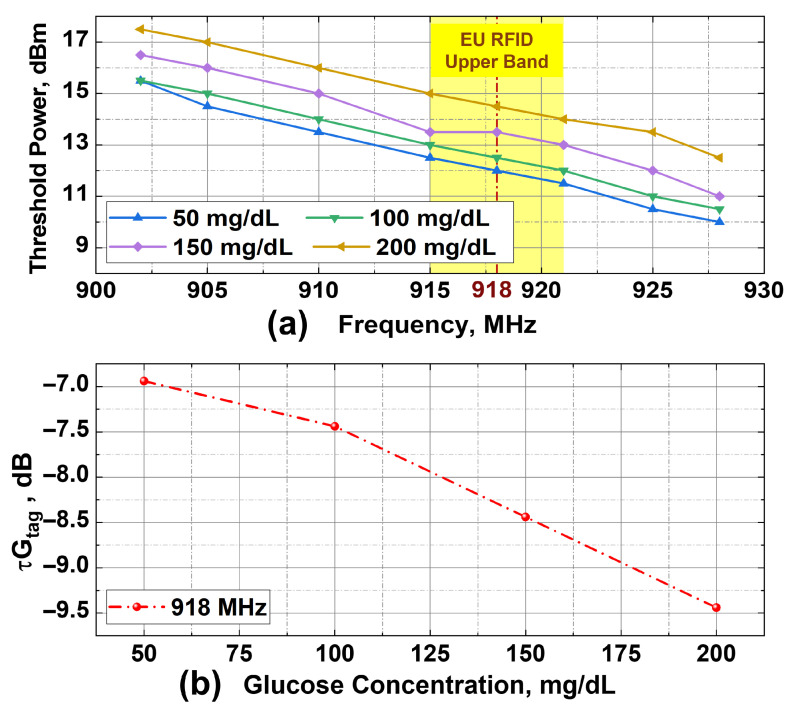
Glucose measurements. (**a**) Threshold power vs. frequency for four glucose concentrations. (**b**) Extracted $\tau G_{\mathrm{tag}}$ values at 918 MHz.

The procedure was repeated four times for each glucose concentration (50, 100, 150, and 200 mg/dL) to ensure reliability of the measurements. Figure 7a presents the average threshold power measured across the four trials as a function of frequency. A general decline in threshold power with increasing frequency is observed, indicating improved antenna response, likely due to reduced reactance mismatch and enhanced gain. Moreover, the threshold power increases monotonically with glucose concentration across nearly all frequencies, indicating the sensor responsiveness to concentration variations. For instance, at 918 MHz, which is the center frequency of the upper European UHF RFID band, a dynamic range of around 2.5 dB is observed in the threshold power between the lowest and highest glucose concentrations.

Furthermore, the measured threshold power values were used to compute the $\tau G_{\mathrm{tag}}$ product, which serves as a key performance indicator introduced in the design concept section. To quantify this response, the product $\tau G_{\mathrm{tag}}$ was extracted at 918 MHz using the following rearranged RFID read range equation:

(6) $$\tau G_{\mathrm{tag}}={\left(\frac{4\pi d}{\lambda }\right)}^{2}\cdot \frac{P_{\mathrm{chip}}}{P_{\mathrm{th}}G_{t}}$$

where *d* is the fixed read distance (0.8 m), $P_{\mathrm{chip}}$ is the chip read sensitivity (in watts), λ is the wavelength at 918 MHz, $P_{\mathrm{th}}$ is the measured threshold power, and $G_{t}$ (7 dBi) is the gain of the reader antenna. Figure 7b shows the extracted $\tau G_{\mathrm{tag}}$ values for each concentration. A decreasing trend is observed, from around −7 dB at 50 mg/dL to −9.5 dB at 200 mg/dL. This reduction reflects the degradation in impedance matching and radiation efficiency caused by increased dielectric loading at higher glucose concentrations, which ultimately affects the read range of the RFID tag.

#### 4.3.2. Read Range Analysis

To validate the sensor performance under practical operating conditions, the read range was measured using the M6E reader configured to operate within the upper European UHF RFID band. For each glucose concentration, the threshold power was recorded across four repeated trials, where the transmit power was progressively reduced from 20 dBm in 0.1 dBm steps until the tag ceased to respond. The use of relatively small power steps in these measurements improves the resolution of the maximum read range and the corresponding estimation of glucose concentration, in accordance with the Friis transmission equation.

**Figure 8:**
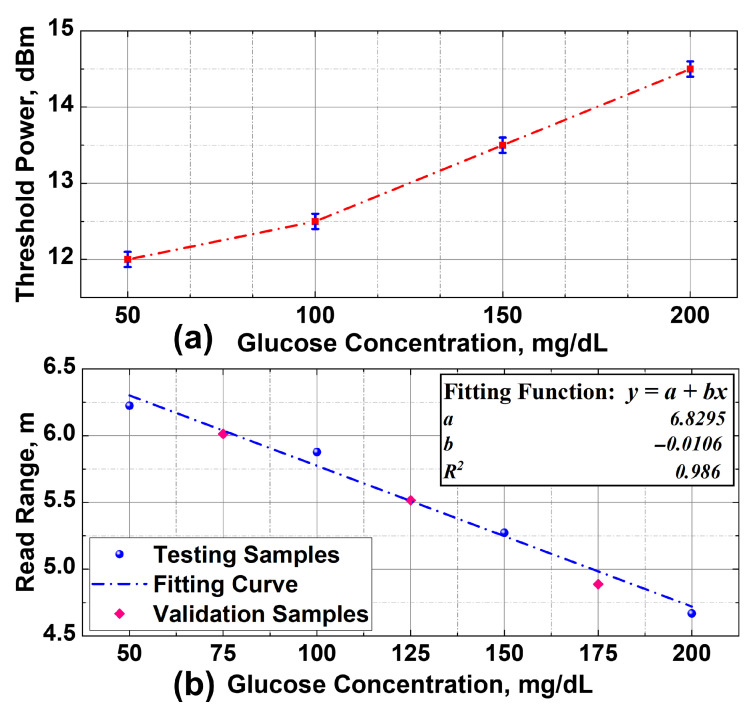
Glucose measurements. (**a**) Threshold power variations for four test concentrations. (**b**) Extracted read range variations for test and validation concentrations with linear curve fitting model.

The averaged threshold power values from these trials were then used to calculate the corresponding read range. Initially, measurements were conducted for test glucose concentrations of 50, 100, 150, and 200 mg/dL. The results are summarized in Table 2, while Figure 8 illustrates the corresponding average threshold power and read range. Notably, the variation in threshold power exhibited a standard deviation below 0.1 dBm across repeated trials, which indicates good measurement consistency and repeatability.

Furthermore, a monotonic decrease in the read range was observed with increasing glucose concentration, as illustrated in Figure 8b. A linear fitting model was applied to quantify this read range behavior, resulting in a high coefficient of determination ($R^{2}=0.986$), which confirms the strong linearity of the sensor response. To further validate the accuracy of this model, additional measurements were carried out using intermediate glucose concentrations of 75, 125, and 175 mg/dL, following the same measurement procedure. It can be observed that the validation points closely follow the fitted curve. Moreover, Table 3 presents a quantitative comparison between the measured and predicted values at these concentrations, showing minimal error. These findings confirm the reliability of the linear fitting model and demonstrate the ability of the sensor to effectively distinguish the varying levels of glucose concentrations in the aqueous solutions.

The sensor can be calibrated prior to deployment using reference glucose solutions of known concentrations to account for potential environmental variations, particularly changes in ambient temperature. This involves placing standard solutions above the CSRR sensing region, recording the corresponding read range, and using this baseline to adjust subsequent measurements. Since the sensor exhibits a linear response, as demonstrated by the high coefficient of determination, a simple two-point calibration is sufficient for linear interpolation. This approach enables field-ready calibration without the need for specialized instruments, aligning well with the sensor’s passive and portable design objectives.

## 5. Discussion and Future Work

Finally, to contextualize the performance of the proposed RFID-CSRR glucose sensor, Table 4 provides a comparative analysis of recent CSRR-based glucose sensing systems reported in the literature. The table summarizes key design parameters, including sensor configuration, sensing parameters, measurement equipment, operating frequency, glucose concentration range, sensitivity, and system portability. As observed, these sensors typically operate between 1 and 6 GHz and rely on reflection or transmission measurements obtained using vector network analyzers (VNAs). Although such instrumentation offers promising sensitivity, it generally restricts these systems to laboratory environments due to the complexity of the setup and cost of the required RF equipment. Moreover, a few alternative approaches employ portable radars or gain/phase detectors for the extraction of sensing parameters, but still depend on specialized RF sources and supporting circuitry, which also limit their practical deployment.

In contrast, the proposed sensor employs a fully passive UHF RFID platform integrated with a CSRR, enabling a battery-free and portable wireless solution that is both practical and directly measurable using commercial RFID readers. It demonstrates a sensitivity of 1.04 cm/(mg/dL) based on read range variations and enables a simplified calibration procedure owing to its linear response to glucose concentrations. This distinction facilitates real-world deployment without requiring specialized laboratory equipment.

While the proposed sensor demonstrates reliable performance under controlled laboratory conditions, certain limitations warrant further investigation. In this study, the tested glucose concentration range of 50–200 mg/dL spans clinically relevant thresholds for hypoglycemia (<70 mg/dL) and hyperglycemia (>180 mg/dL); however, future work will aim to expand this detection range. In addition, instead of aqueous glucose solutions, the robustness of the sensor will be validated in biologically relevant media such as plasma or serum, where additional dielectric dispersion and conductivity effects may arise. Furthermore, practical deployment scenarios will involve portable RFID readers measuring read range dynamically with proper orientation, rather than relying on the fixed-distance setup used in this study. Despite this, the proposed RFID-CSRR sensor presents a compelling proof of concept that combines portability, reliable sensitivity, and ease of measurement, making it well suited for concentration monitoring applications and effectively addressing the key limitations of existing CSRR-based dielectric sensing techniques.

## 6. Conclusions

This paper presented a passive UHF RFID-based glucose sensor that integrates a circular CSRR structure with an H-slot antenna for dielectric-based concentration sensing in aqueous glucose solutions. By combining the high dielectric sensitivity of CSRRs with the wireless and battery-free operation of UHF RFID technology, the proposed design enables a fully passive and portable sensing platform. The sensor operates within the FCC-authorized UHF RFID band and uses read range variation as a practical sensing parameter. Full-wave simulations and experimental measurements demonstrated a monotonic decrease in read range with increasing glucose concentration. Compared to existing CSRR-based glucose sensors that rely on laboratory RF instrumentation, the proposed system eliminates the need for complex equipment and supports real-time measurements using commercially available portable RFID readers. These results demonstrate the feasibility and practical advantages of integrating high-sensitivity resonators with passive RFID technology for dielectric-based concentration monitoring, with future work focused on extending the platform to broader applications in liquid characterization and chemical sensing.

## Figures and Tables

**Table 1:** Optimized geometrical parameters of the H-slot antenna and CSRR for the proposed glucose concentration sensor.

H-Slot Antenna		CSRR Resonator	
Parameter	Value (mm)	Parameter	Value (mm)
Patch length (*L*)	72.3	Inner ring radius ($R_{1}$)	11.5
Patch width (*W*)	72.3	Outer ring radius ($R_{2}$)	23.3
Slot length ($L_{1}$)	27.0	Inner ring gap ($G_{1}$)	2.0
Slot width ($W_{1}$)	23.2	Outer ring gap ($G_{2}$)	4.0
Feedline width ($W_{2}$)	2.56	Inner ring width ($W_{3}$)	2.0
Feed point gap ($C_{1}$)	0.50	Outer ring width ($W_{4}$)	5.0

**Table 2:** Test and validation results of RFID-CSRR-based glucose concentration sensor.

Measurements	Test Concentrations (mg/dL)				Validation Concentrations (mg/dL)		
	50	100	150	200	75	125	175
First read power (dBm)	12.0	12.5	13.5	14.4	12.3	13.0	14.0
Second read power (dBm)	12.1	12.4	13.4	14.6	12.4	13.1	14.1
Third read power (dBm)	11.9	12.6	13.6	14.5	12.2	13.0	14.2
Fourth read power (dBm)	12.0	12.5	13.5	14.5	12.3	13.1	14.1
Average read power (dBm)	12.0	12.5	13.5	14.5	12.3	13.05	14.1
Maximum read range (m)	6.225	5.877	5.273	4.668	6.013	5.516	4.888

**Table 3:** Error between measured validation points and curve-fitted read range.

Read Range (m)	Validation Concentrations (mg/dL)		
	75	125	175
Validation points	6.013	5.516	4.888
Linear fit	6.035	5.505	4.975
|Error|	0.022	0.011	0.087

**Table 4:** Comparison of CSRR-based glucose concentration sensors.

Ref.	Sensor Configuration	Sensing Parameters	Measurement Equipment	Frequency (GHz)	Range (mg/dL)	Sensitivity	Portability
[22]	Circular CSRR coupled with stripline structure	$|S_{11}|$	VNA	1.0–6.0	0–110	0.0345 dB/(mg/dL)	Laboratory
[19]	Hexagonal CSRR with interdigital capacitor	$|S_{11}|$ and $f_{r}$	VNA	1.0–5.0	0–150	0.023 dB/(mg/dL) 1.73 MHz/(mg/dL)	Laboratory
[17]	Double-square CSRR (45°) with microstrip line	$|S_{21}|$	VNA	1.0–4.0	40–500	0.0056 dB/(mg/dL)	Laboratory
[30]	Hexagonal CSRR array with microstrip line	$f_{r}$	VNA or radar module	2.4–2.5	70–120	≈0.94 MHz/(mg/dL)	Semi-Portable
[45]	Circular CSRR as DGS with microstrip line	$|S_{11}|$ and $f_{r}$	VNA	2.0–6.0	0–250	0.0406 dB/(mg/dL) 0.117 MHz/(mg/dL)	Laboratory
[24]	Dual-square CSRRs with switching circuit	$|S_{21}|$	VNA + FG	2.45	0–400	0.008 dB/(mg/mL)	Laboratory
[11]	Square CSRR with open-ended line	$|S_{11}|$ and $f_{r}$	VNA	2.48	0–500	0.5 dB/(mg/mL) ≈1.24 MHz/(mg/mL)	Laboratory
[46]	Triple-pole CSRR with microstrip line	$|S_{11}|$ and $|S_{21}|$	VNA	1.0–6.0	70–120	$|S_{11}|$: 3.45 dB/(mg/dL) $|S_{21}|$: 6.2 dB/(mg/dL)	Laboratory
[47]	CSRR-loaded T-patch antenna	$|S_{11}|$	VNA	1.02–2.24	0–600	0.0016 dB/(mg/dL)	Laboratory
[32]	Modified CSRR with differential SIW	DC voltages	RF generator & detectors	1.674	0–300	$V_{\mathrm{mag}}$: 3.05 mV/(mg/dL) $V_{\mathrm{phs}}$: 4.21 mV/(mg/dL)	Laboratory
This Work	Circular CSRR with H-slot UHF RFID antenna	Read range	UHF RFID reader	0.902–0.928	50–200	1.04 cm/(mg/dL) $d_{\max}$: 6.23–4.67 m	Fully Portable

VNA: vector network analyzer, FG: function generator, $f_{r}$: resonance frequency.

## Data Availability

Data are contained within the article.
